# Imipramine Administration in *Brucella abortus 2308*-Infected Mice Restores Hippocampal Serotonin Levels, Muscle Strength, and Mood, and Decreases Spleen CFU Count

**DOI:** 10.3390/ph16111525

**Published:** 2023-10-27

**Authors:** José Luis Maldonado-García, Gilberto Pérez-Sánchez, Enrique Becerril-Villanueva, Samantha Alvarez-Herrera, Lenin Pavón, Luvia Sánchez-Torres, Gabriel Gutiérrez-Ospina, Manuel Iván Girón-Pérez, Gabriela Damian-Morales, Jesús Octavio Maldonado-Tapia, Rubén López-Santiago, Martha C. Moreno-Lafont

**Affiliations:** 1 Laboratorio de Psicoinmunología, Dirección de Investigaciones en Neurociencias del Instituto Nacional de Psiquiatría Ramón de la Fuente Muñiz, Mexico City 14370, Mexico; joselmgarci@comunidad.unam.mx (J.L.M.-G.); gilberto.perez.sanchez@inprf.gob.mx (G.P.-S.); lusenbeve@inprf.gob.mx (E.B.-V.); dra.alvarezherrera@gmail.com (S.A.-H.); jmaldonadot1200@egresado.ipn.mx (J.O.M.-T.); 2 Laboratorio de Inmunología Celular, Departamento de Inmunología, Escuela Nacional de Ciencias Biológicas, Instituto Politécnico Nacional, Mexico City 07738, Mexico; damianunam@hotmail.com (G.D.-M.); rlopezsantiago@hotmail.com (R.L.-S.); 3 Laboratorio de Inmunología de los Microorganismos, Departamento de Inmunología, Escuela Nacional de Ciencias Biológicas, Instituto Politécnico Nacional, Mexico City 07738, Mexico; luviasanchez@hotmail.com; 4 Laboratorio de Biología de Sistemas, Departamento de Biología Celular y Fisiología, Instituto de Investigaciones Biomédicas y Coordinación de Psicobiología y Neurociencias, Facultad de Psicología, Universidad Nacional Autónoma de México, Mexico City 04510, Mexico; gabo@biomedicas.unam.mx; 5 Laboratorio Nacional LANIIA-Nayarit, Universidad Autónoma de Nayarit, Tepic 63173, Mexico; ivangiron@uan.edu.mx

**Keywords:** brucellosis, imipramine, serotonin, strength test, IL-6, physical performance

## Abstract

Brucellosis infection causes non-specific symptoms such as fever, chills, sweating, headaches, myalgia, arthralgia, anorexia, fatigue, and mood disorders. In mouse models, it has been associated with increased levels of IL-6, TNF-α, and IFN-γ, a decrease in serotonin and dopamine levels within the hippocampus, induced loss of muscle strength and equilibrium, and increased anxiety and hopelessness. Imipramine (ImiP), a tricyclic antidepressant, is used to alleviate neuropathic pain. This study evaluated the effects of ImiP on Balb/c mice infected with *Brucella abortus 2308* (Ba) at 14- and 28-days post-infection. Serum levels of six cytokines (IFN-γ, IL-6, TNF-α, IL-12, MCP-1. and IL-10) were assessed by FACS, while the number of bacteria in the spleen was measured via CFU. Serotonin levels in the hippocampus were analyzed via HPLC, and behavioral tests were conducted to assess strength, equilibrium, and mood. Our results showed that mice infected with *Brucella abortus 2308* and treated with ImiP for six days (Im6Ba14) had significantly different outcomes compared to infected mice (Ba14) at day 14 post-infection. The mood was enhanced in the forced swimming test (FST) (*p* < 0.01), tail suspension test (TST) (*p* < 0.0001), and open-field test (*p* < 0.0001). Additionally, there was an increase in serotonin levels in the hippocampus (*p* < 0.001). Furthermore, there was an improvement in equilibrium (*p* < 0.0001) and muscle strength (*p* < 0.01). Lastly, there was a decrease in IL-6 levels (*p* < 0.05) and CFU count in the spleen (*p* < 0.0001). At 28 days, infected mice that received ImiP for 20 days (Im20Ba28) showed preservation of positive effects compared to infected mice (Ba28). These effects include the following: (1) improved FST (*p* < 0.0001) and TST (*p* < 0.0001); (2) better equilibrium (*p* < 0.0001) and muscle strength (*p* < 0.0001); (3) decreased IL-6 levels (*p* < 0.05); and (4) reduced CFU count in the spleen (*p* < 0.0001). These findings suggest the potential for ImiP to be used as an adjuvant treatment for the symptoms of brucellosis, which requires future studies.

## 1. Introduction

For at least a decade, research has suggested that treatment-resistant mood disorders occur secondarily to microbial infections, either through direct neural infection or chronic neuroinflammation as a mediator [1,2,3,4,5,6,7,8]. Microbes can enter the body from new or internal sources when mucosal integrity is compromised and commensal/symbiotic organisms that inhabit the body gain access to its internal milieu [3,9,10,11]. On the other hand, it has also been proposed that mood disorders, especially depression, provide an evolutionary advantage to hosts in coping with microbial disease by providing reciprocal modulatory interactions between mood and immune response (the pathogen–host defense theory of depression) [12,13]. In contrast, it has also been observed that depression may predispose an individual to developing severe infections [14,15] and/or worsen disease progression and outcome [12,13,16]. In this context, it is fundamental to enhance psychoimmune resilience [17] by modulating depression in already-infected individuals. Our group has previously reported the existence of neuroimmunoendocrine and behavioral alterations in a murine model of brucellosis [18], a zoonosis found in Latin America, Africa, and the Mediterranean region. In humans, this infection causes non-specific symptoms such as fever, chills, sweats, headache, myalgia, arthralgia, anorexia, fatigue, weight loss, lymphadenopathy (10–20%), splenomegaly (20–30%), diarrhea [19], anxiety, and other neurological symptoms [20,21]. In our murine model of brucellosis infection, the presence of anxiety and depression-like symptoms, altered serotonin levels in the hippocampus, and increased peripheral inflammation have been observed [18]. Although there are pharmacological treatments for brucellosis, the infection remains a widespread disease. The administration of antibiotics alone or in combination has not been successful in the treatment of human brucellosis, and this, together with the presence of relapses and lack of adherence to therapy, contributes to the chronic development of this disease, which has a cosmopolitan distribution [22,23,24]. Therefore, this experimental mouse model seems useful in exploring whether down-modulation of depression could contribute to improve the clinical outcome in *Brucella*-infected mice using imipramine (ImiP). This is a tricyclic antidepressant with serotonin transporter blocking activity, which allows for the restoration of serotonin levels in the hippocampus and behavioral symptoms with an immunomodulatory effect [25,26]. Previously, it has been reported that ImiP can limit *Leishmania donovani* infection in animal models [27,28,29] and may have direct regulatory effects on pro-inflammatory cytokine synthesis and release. Tricyclic antidepressants such as ImiP have been shown to decrease the release of pro-inflammatory cytokines such as IL-6, IL-1β, and TNF-α in human blood monocytes and IL-2 and IFN-γ in T cells in vitro [30,31,32]. Such a decrease in release may follow an increase in intracellular levels of cAMP [30,31,32] and/or a decreased availability of intracellular serotonin in phagocytic cells [33,34,35,36,37]. In addition, increases in extracellular serotonin concentrations after ImiP administration could negatively affect the mRNA transcription of pro-inflammatory cytokines [33,34,35,36,37]. The aim of this work was to evaluate the beneficial effects of ImiP administration in a murine model of *B. abortus 2308* infection for the treatment of brucellosis-associated symptoms.

## 2. Results

**Figure 1:**
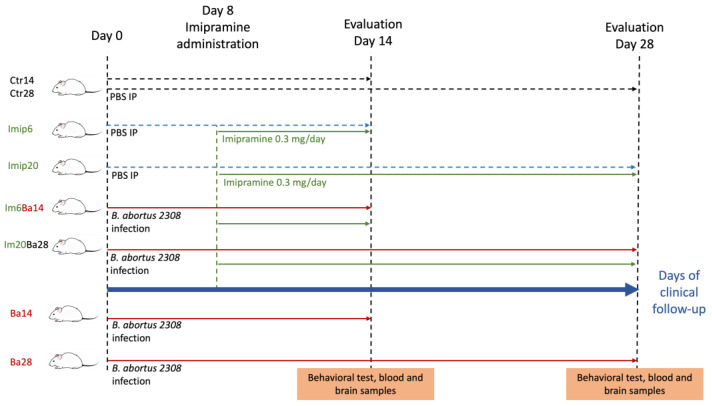
Experimental design.

Eight groups receiving the following treatments were evaluated in this study (Figure 1): PBS (referred to as Ctr14 and Ctr28); infection with *Brucella abortus 2308* (referred to as Ba14 and Ba28); infection with *B. abortus 2308* and treated with imipramine (referred to as Im6Ba14 and Im20Ba28); and a group treated with imipramine only (referred to as ImiP6 and ImiP20) (for more details please refer to the Section 4).

### 2.1. Orogastric Administration of Imipramine Restores Mood and Hippocampal Serotonin Levels in Im6Ba14 and Im20Ba28 Mice

**Figure 2:**
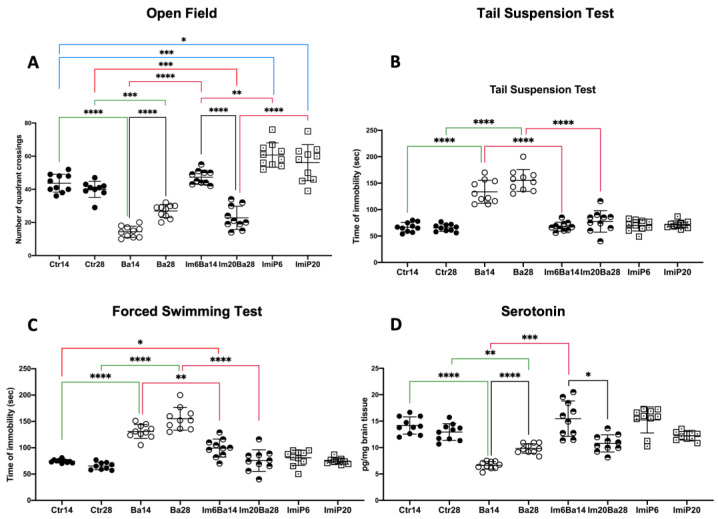
Behavioral tests and serotonin quantification. (**A**) Results of the open-field test, where the number of frame changes was quantified; an increase in the number of changes represents the presence of anxiety. (**B**) Results of the tail suspension test, which quantifies immobility time in seconds; a longer immobility time can be interpreted as greater hopelessness. (**C**) Results of the forced swim test, where immobility time was quantified in seconds; a longer immobility time can be interpreted as greater hopelessness. (**D**) Results of serotonin quantification in the hippocampus. The scatter plot shows the course of hippocampal serotonin concentrations on days 14 and 28 of the follow-up in control mice (Ctr14 and Ctr28), ImiP mice (ImiP6 and ImiP20), *B. abortus 2308*-infected mice (Ba14 and Ba28), and ImiP-treated *B. abortus 2308*-infected mice (Im6Ba14 and Im20Ba28). Orogastric administration of ImiP to infected mice (ImBa groups) improved depression-like conditions, reduced anxiety, and restored hippocampal serotonin levels by day 14 of follow-up. Depression was ameliorated by day 28 of follow-up. Statistical significance is shown as follows: * = *p* < 0.05; ** = *p* < 0.01; *** = *p* < 0.001; **** = *p* < 0.0001. Scatter plots show mean ± SD.

The mean ± standard deviation (SD) of the behavioral test results and hippocampal serotonin levels for each group are shown in Figure 2. Statistical analysis showed differences between the study groups in the open-field test (OF) (F* = 67.09, df = (7.000, 42.69), *p* < 0.0001), forelimb grip strength test (FST) (W = 43.51, df = (7.000, 28.62), *p* < 0.0001), tail suspension test (TST) (W = 32.55 df = (7.000, 30.55), *p* < 0.0001), and serotonin levels (W = 59.55 df = (7.000, 30.40), *p* < 0.0001). In FST and TST, Ba14 and Ba28 mice exhibited increased immobility time compared to the corresponding Ctr group throughout the follow-up period (TST 133.70 ± 21.74 vs. 66.90 ± 8.81 *p* < 0.0001 on day 14 and 155.20 ± 20.71 vs. 65.60 ± 6.80 on day 28 *p* < 0.0001 and FST 130.90 ± 13.23 vs. 74.40 ± 3.20 on day 14 and 155.00 ± 21.56 vs. 65.33 ± 7.08 *p* < 0.0001). No differences in immobility time were found when comparing Ctr14 vs. Imip6 and Ctr 28 vs. ImiP20. Taken together, these results suggest that Ba14 and Ba28 mice exhibit a lack of motivation (i.e., depression-like behavior) [18]. Reduced hippocampal serotonin levels are often associated with low motivation in mice, and Ba14 and Ba28 mice exhibited significantly reduced hippocampal serotonin levels compared to Ctr14 and Ctr28, respectively, throughout the follow-up period (6.62 ± 0.71 vs. 14.18 ± 1.62 *p* < 0.0001 on day 14 and 9.83 ± 0.90 vs. 12.93 ± 1.58 *p* < 0.01 on day 28). At the same time, no differences were found when comparing ImiP6 vs. Ctr14 and ImiP20 vs. Ctr28. In contrast to the Ba groups, Im6Ba14 and Im20Ba28 mice significantly improved their FST scores compared to the Ba groups on day 14 (130.90 ± 13.23 vs. 99.40 ± 17.09 *p* < 0.01) and on day 28 (155.00 ± 21.56 vs. 75.60 ± 20.60 *p* < 0.0001). A similar phenomenon is observed in the TST, where the ImBa groups improved their scores compared to the Ba groups (67.60 ± 8.43 vs. 133.70 ± 21.74 *p* < 0.0001 at day 14 and 77.60 ± 20.30 vs. 155.20 ± 20.71 *p* < 0.0001 at day 28). In the hippocampus, serotonin concentrations were improved at day 14 in Im6Ba14 compared to Ba14 (6.62 ± 0.71 vs. 15.46 ± 3.35 *p* < 0.001), and although no significant differences in serotonin levels were detected between Im20Ba28 and Ba28, the TST and FST parameters are equivalent to the values presented by the Ctr groups. Thus, our results show that administration of ImiP to Ba-infected mice (ImBa groups) improves hippocampal serotonin levels and depression-like behavior. The OF anxiety test showed that the ImiP groups exhibited increased exploratory behavior at day 14 (60.70 ± 7.30 vs. 43.70 ± 5.39 *p* < 0.001), although the intensity of the phenomenon decreased by day 28 (56.20 ± 10.88 vs. 40.00 ± 4.87 *p* < 0.05) compared to Ctr14 and Ctr28, respectively. As previously published, infected Ba mice exhibited decreased exploratory behavior (14.40 ± 3.37 vs. 43.70 ± 5.39, *p* < 0.0001 at day 14) compared to their Ctr counterparts. Im6Ba14 mice showed exploratory behavior equivalent to that of their Ctr14 at day 14, better than that of Im20Ba28 mice at the end of the follow-up (22.80 ± 7.00 vs. 40.00 ± 4.87 *p* < 0.0001). Nevertheless, the Im20Ba28 group showed an exploratory behavior like that of Ba28, lower than that of controls (22.80 ± 7.00 vs. 40.00 ± 4.87 *p* < 0.001) and ImiP 20 (22.80 ± 7.00 vs. 56.20 ± 10.88 *p* < 0.0001). Thus, the effect of ImiP is likely to improve anxious behavior in infected mice in the short term. The mean ± SD values obtained from the analyses of the mice can be found in the Supplementary Materials, Table S1.

### 2.2. Orogastric Administration of Imipramine Restores Equilibrium and Muscular Strength in ImBa Mice

**Figure 3:**
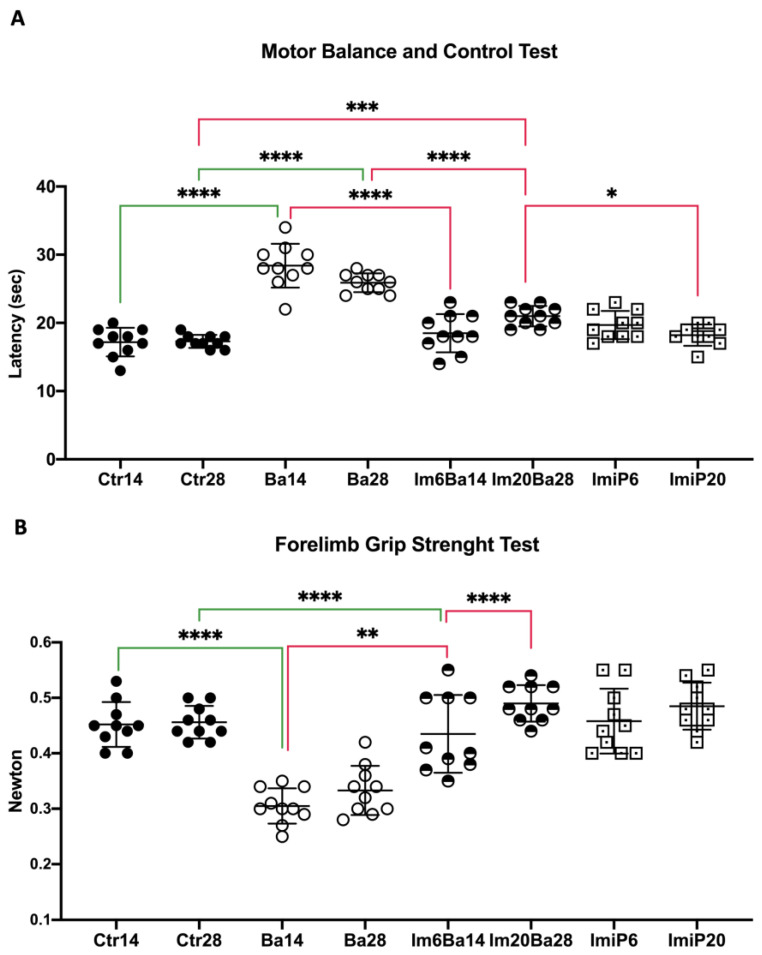
Assessment of motor disabilities, muscle weakness. (**A**) The motor balance and control test measure the time it takes the mouse to perform the test; a long time can be interpreted as a motor disability. (**B**) The forelimb grip strength test evaluates a mouse’s muscular endurance in Newtons; a lower score on the test indicates an increase in muscle weakness. Scatter plots show the mean ± SD of the values obtained in motor performance as an index of body condition at days 14 and 28 of follow-up in control mice (Ctr14 and Ctr28), ImiP mice (ImiP6 and ImiP20), *B. abortus 2308*-infected mice (Ba14 and Ba28), and ImiP-treated *B. abortus 2308*-infected mice (Im6Ba14 and Im20Ba28). Orogastric administration of ImiP significantly improved the body condition of Im6Ba14 and Im20Ba28 mice. Statistical significance is shown as follows: * = *p* < 0.05. ** = *p* < 0.01; *** = *p* < 0.001; **** = *p* < 0.0001.

Statistical differences were found in the motor balance and control test (MBCT) (W = 46.90 df: 7.000, 30.01, *p* < 0.0001) and the forelimb grip strength test (FGST) (W = 33.35 df: 7.000, 30.71, *p* < 0.0001). As previously reported [18], our results showed an increase in MBCT latency when comparing Ba14 with Ctr14 (28.40 ± 3.20 vs. 17.20 ± 2.09 *p* < 0.0001) and Ba 28 with Ctr28 (25.90 ± 1.37 vs. 17.30 ± 0.94 *p* < 0.0001), while the presence of muscle weakness in the FGST was also higher during clinical follow-up when comparing infected mice with control mice (Ba14: 0.30 ± 0.03 vs. 0.45 ± 0.04 *p* < 0.0001; Ba28 0.33 ± 0.04 vs. 0.45 ± 0.02 *p* < 0.0001) (Figure 3). The ImiP groups did not differ from the corresponding controls in terms of equilibrium and muscle strength. Furthermore, intra-gastric administration of ImiP on days 8–14 or 8–28 post-infection significantly improved the MCTB score in Im6Ba14 (18.50 ± 2.79 vs. 28.40 ± 3.20 *p* < 0.0001) and Im20Ba28 (21.00 ± 1.49 vs. 25.90 ± 1.37 *p* < 0.0001) mice compared to Ba14 and Ba28 groups, respectively. Similarly, imipramine treatment improved muscle strength in mice in the Im6Ba14 (0.43 ± 0.07 vs. 0.30 ± 0.03 *p* < 0.01) and Im20Ba28 (0.49 ± 0.03 vs. 0.33 ± 0.04 *p* < 0.0001) groups compared with Ba14 and Ba28, respectively, so that their performance was equivalent to that of controls and much better than that observed in Ba. Thus, our results suggest that the administration of ImiP to *B. abortus 2308*-infected mice can help restore balance and muscle strength. The mean ± SD values obtained from the analyses of the mice can be consulted in the Supplementary Materials, Table S2.

### 2.3. Imipramine Administration Decreases IL-6 Serum Levels in ImBa Mice Group

In our results, we obtained concentrations below the detection limit for MCP-1 and IL-10 in all groups; therefore. they were declared as not detected (ND) and were not published. In the same way, we obtained values below the detection limit in the Ctr, ImiP6 and ImiP20 groups for IL-6, IL-12, and IFN-γ, which were also declared as ND; in all cases, these values were not considered in the statistical analysis (Table 1). A significant difference in TNF-α levels (F* = 112.1, df (7.000, 29.37) *p* < 0.0001) was found, and no changes in circulating levels were detected via imipramine administration (Crt vs. ImiP), while Im6Ba20 mice showed significantly lower circulating levels (*p* < 0.01)—similar to the Im20Ba28 group (*p* < 0.0001)—compared to Ba mice groups. Im20Ba28 mice showed a significantly greater decrease in serum TNF-α levels than Im6Ba14 mice (*p* < 0.0001). In both cases, TNF-α levels did not return to baseline levels when compared to the Ctr groups (Im6Ba14 vs. Ctr14 *p* < 0.0001 and Im20Ba28 vs. Ctr28 *p* < 0.0001). In the case of IL-6 (W = 113.0, df (3.000, 21.75) *p* < 0.0001), IL-12 (W = 6.017, df (3.000, 17.11) *p* < 0.01), and IFN-γ (W = 175.8, df (3.000, 19.27) *p* < 0.01), the Ba and ImBa groups were compared to determine whether imipramine treatment modifies cytokine concentrations. No changes were found in the serum concentration of IL-12 and IFN-γ when Ba and ImBa groups were compared; however, a decrease in the concentration of IL-6 was observed when comparing the Im6Ba14 and Ba14 groups (*p* < 0.0001), while at day 28, IL-6 was ND in the Im20Ba28 group (see Table 1).

### 2.4. Treatment with Imipramine Did Not Modify the Number of Macrophages and Dendritic Cells in the Spleen of Infected Animals

**Figure 4:**
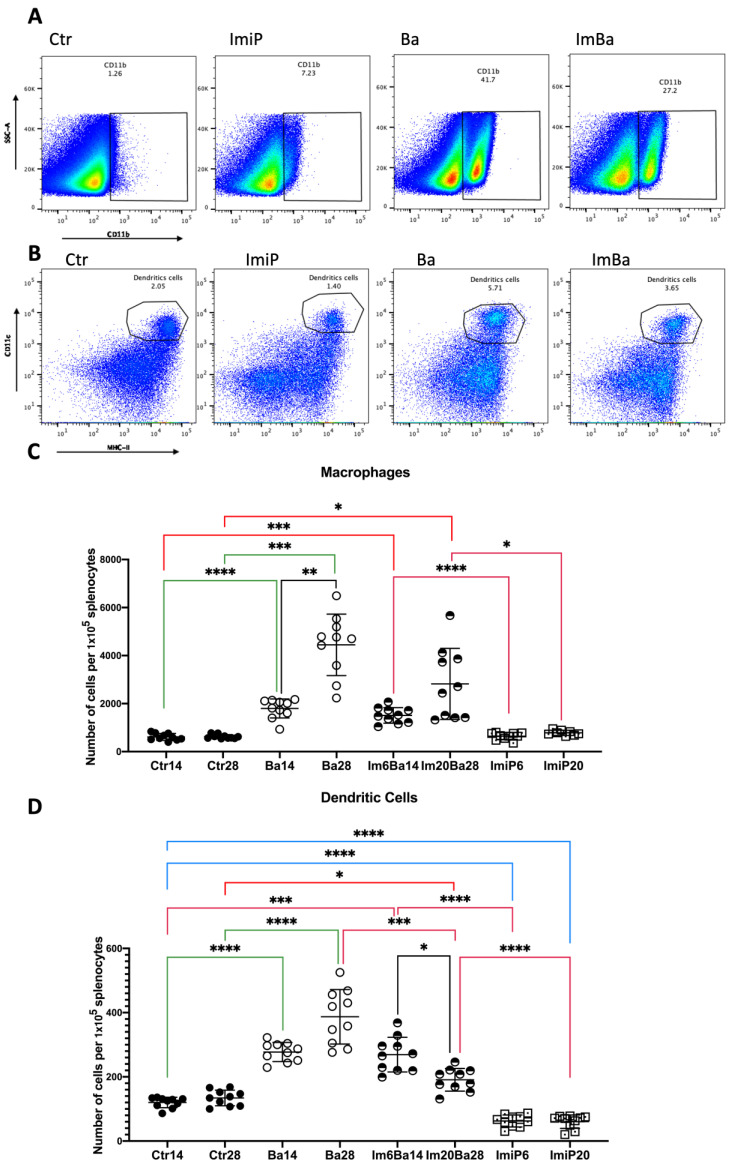
Macrophages and dendritic cell quantification in the spleen. (**A**,**B**) show representative dot plots of flow cytometric analysis of macrophages and dendritic cells in the analyzed groups. Dot plots show the percentage of macrophages or dendritic cells per total number of events analyzed (approximately 3 × 10^6^). (**C**,**D**) show the absolute number of macrophages (**C**) and dendritic cells (**D**) adjusted to the number of cells per 1 × 10^5^ splenocytes at 14 and 28 days in control mice (Ctr14 and Ctr28), ImiP mice (ImiP6 and ImiP20), *B. abortus 2308*-infected mice (Ba14 and Ba28), and ImiP-treated *B. abortus 2308*-infected mice (Im6Ba14 and Im20Ba28). ImBa mice vs. Ba mice showed no differences. Statistical significance is shown as follows: * = *p* < 0.05. ** = *p* < 0.01; *** = *p* < 0.001; **** = *p* < 0.0001.

Statistical differences were found in the number of macrophages (F* = 35.77, df = (7.000, 20.64) *p* < 0.0001) and dendritic cells (F* = 76.91, df = (7.000, 27.40) *p* < 0.0001) in the spleen of the studied groups. As expected during active infection, an increase in splenic macrophage numbers was found in the Ba14 and Ba28 groups of macrophages compared to Ctr14 and Ctr28 (14: 1797.00 ± 396.60 vs. 612.80 ± 144.30 *p* < 0.0001 at day 14; and 4446.00 ± 1279.00 vs. 624.20 ± 85.19 *p* < 0.001 at day 28) and dendritic cells (277.30 ± 29.94 vs. 120.10 ± 16.35 *p* < 0.0001 at day 14; and 28: 387.00 ± 84.94 vs. 134.30 ± 24.36 *p* < 0.0001 at day 28) (Figure 3). When comparing the Im6Ba14 group with the Ba14 group, as well as Im20Ba28 with Ba28, no statistically significant differences were found in splenic macrophage and dendritic cell counts, suggesting that imipramine treatment does not alter the macrophage and dendritic cell counts in the infection (Figure 4). It is important to note that healthy animals receiving ImiP did not show any numerical changes in macrophages, although dendritic cells showed a significant decrease in their number throughout the ImiP6 (62.60 ± 18.73 vs. 120.10 ± 16.35 *p* < 0.0001) and ImiP20 (60.40 ± 20.80 vs. 134.30 ± 24.36 *p* < 0.0001) groups when compared to the respective control group. The mean ± SD values obtained from the analyses of the mice can be found in the Supplementary Materials, Table S3.

### 2.5. After Treatment with Imipramine, Mice in the ImBa Group Had Lower Spleen CFU Counts

**Figure 5:**
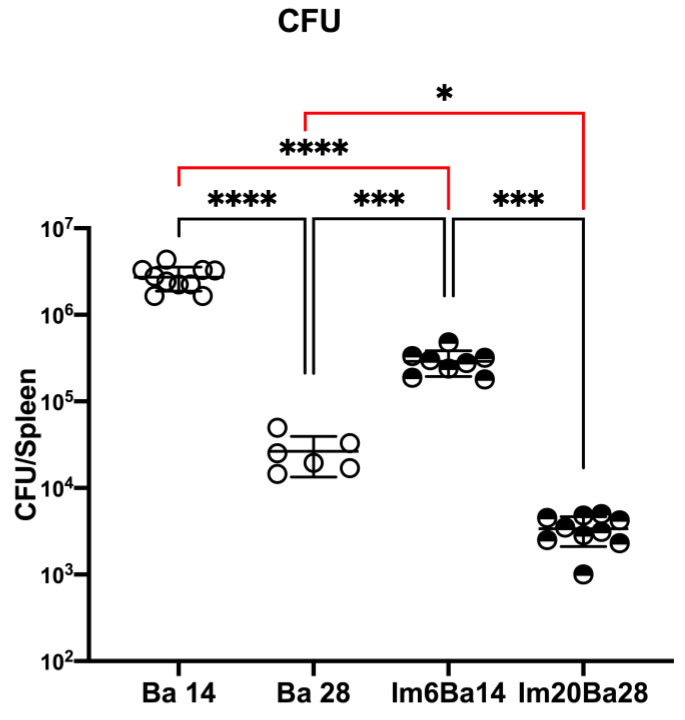
*Brucella abortus 2308* CFU in the spleen. The plots of the data are presented in Log10. The scatter plot shows the mean ± SD of *B. abortus 2308* colony-forming units (CFU) derived from individual spleens of *B. abortus 2308*-infected mice (Ba14 and Ba28) and ImiP-treated *B. abortus 2308*-infected mice (Im6Ba14 and Im20Ba28) collected on day 14 and 28. Statistical significance is shown as follows: * = *p* < 0.05; *** = *p* < 0.001; **** = *p* < 0.0001.

Our results showed statistical differences in the number of CFU in the spleen (F* = 95.00; df = (3.000, 9.3) *p* < 0.0001). The Im6Ba14 group had a lower number of *B. abortus 2308* CFU in the spleen when compared to the Ba14 group (289,706 ± 95,787 vs. 2,720,460 ± 845,314 *p* < 0.0001) and Im20Ba28 vs. the Ba28 group (3377 ± 1272 vs. 26,457 ± 13,078 *p* < 0.05), as shown in Figure 5. This finding is particularly interesting because it suggests an ImiP-mediated immunostimulatory effect to eliminate *B. abortus 2308*, as in the work of Mukherjee [27,28]. It is worth noting that ImiP was previously reported to have no antibiotic effect on *Brucella* [38]. We are aware that in the future, we will need to investigate how ImiP affects the CFU count in our model. The mean ± SD values obtained from the analyses of the mice can be consulted in the Supplementary Materials, Table S4.

## 3. Discussion

Brucellosis is a debilitating and disabling disease that causes severe chronic pain, anxiety, and depression, affecting the quality of social and work life lives [39]. Antibiotic treatment of brucellosis has a high relapse rate [24], which is one of the reasons why this zoonosis rapidly becomes chronic in humans and has an economic impact in countries where the disease is endemic [40,41].

Our results show that *Brucella abortus 2308* infection in mice induced a significant increase in circulating levels of IL-6, IL-12, TNF-α, and IFN-γ; an increase in the number of macrophages and dendritic cells in the spleen; a decrease in hippocampal serotonin levels by day 14; and loss of physical strength, balance, and hopelessness, as previously reported by our group [18]. Changes in hippocampal serotonin levels and behavioral changes are because of bacterial antigens, such as lipopolysaccharide, and inflammatory molecules, i.e., IL-1β, TNF-α, and IL-6, and their synergistic effect with other inflammatory mediators [42]. The intensity of the inflammatory response and the relative abundance of bacterial antigens are then associated with neurochemical and behavioral changes [18].

Depression may be a chronic outcome of infectious diseases [1,2,3,4,5,6,7,8]. It may also increase the individual’s risk to develop severe infections [12,13,14,15,16]. Therefore, in clinical settings, reducing depression may help infected patients to successfully resolve microbial infections, even though a depressive tone seems to provide evolutionary advantages (e.g., resource scarcity for microbial invaders) to hosts while fighting infectious diseases [12,13]. Consistent with this statement, in this work, we have shown that ImiP treatment of Brucella-infected (ImBa) mice restores near-normal motivation, increases hippocampal serotonin availability and serum IL-6 levels, and improves balance and muscle strength.

The mechanisms by which ImiP improves motivation are fairly well understood. It increases extracellular concentrations of neurotransmitters after inhibiting serotonin, norepinephrine, and dopamine reuptake [43,44,45,46], a circumstance that improves mood. Brucellosis can lead to the establishment of depression in susceptible individuals; one possible mechanism of this phenomenon is a sustained activation of the inflammatory response that alters the metabolic consumption of tryptophan toward the kynurenine pathway instead of the serotonin pathway [47,48,49]. This mechanism is not unique to brucellosis and has been described in several chronic inflammatory conditions, both in animal models of infection [50,51] and in humans [52,53]. In contrast, the condition of Im6Ba14 and Im20Ba28 mice could be improved by decreasing IL-6 as elevations of this cytokine sensitize pain pathway and producing hyperalgesia through various mechanisms [54,55]. In addition, ImiP consumption is known to reduce circulating levels of IL-6 [25,56].

**Figure 6:**
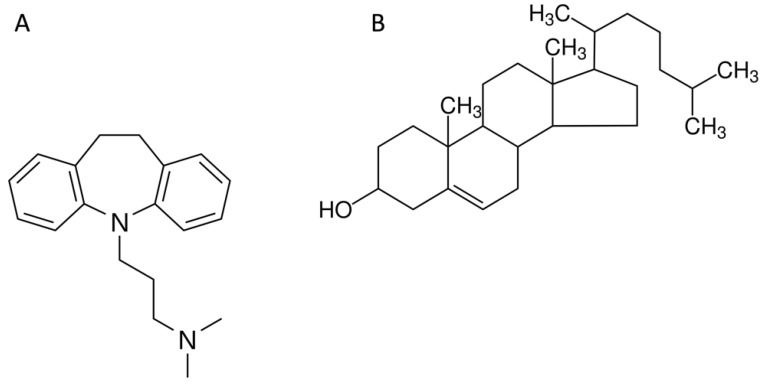
Chemical structures of imipramine and cholesterol. Two-dimensional schematic representation of imipramine (**A**) and cholesterol (**B**) showing their structural similarities.

Our results suggest that ImiP administration in ImBa mice modulates some aspects of the immune response, as described previously [28]. Accordingly, in our experimental series, ImiP treatment reduced bacterial counts in the spleen of infected mice. Previous in vitro reports have shown that monocytes derived from donor PBMC and exposed to ImiP 100 μmol/L exhibit high phagocytic activity after three days of incubation [57]. This could be explained by the fact that macrophages increase their phagocytic activity after ImiP treatment in vitro and in vivo due to their molecular similarity to cholesterol [27,28,58] (Figure 6). ImiP may mimic the effects of cholesterol on signal transduction pathways that promote phagocytosis, immunological synapse formation, and antigen presentation in professional phagocytes [27,28,29,59]. It may also reduce membrane fluidity, which is known to enhance phagocytosis [28]. Previous work suggests that ImiP induces superoxide and nitric oxide production by macrophages in vitro [60,61] and in vivo [27,28,29]. Intracellular cholesterol trafficking is essential for *B. abortus 2308* to consolidate infection in mice. *B. abortus 2308* traverses the host cell membrane using the VirB complex and lipid rafts [62]. However, the insertion of lipid rafts into cell membranes depends on cholesterol transfer from the endosomal compartment to the cell membrane. Cholesterol substitution is likely to be an ImiP mechanism that causes lower CFU counts and could interfere with the integration of lipid rafts into cell membranes. This prevents *B. abortus 2308* from entering target cells [63], making it more susceptible to elimination via phagocytosis. In addition, ImiP has been reported to inhibit macropinocytosis in dendritic cells and macrophages [64]. Macropinocytosis is essential for Brucella infection and replication [65], so it could reduce the infectious capacity of Brucella and contribute to the reduction in CFU obtained from the spleen of infected mice treated with ImiP. It is important to note that ImiP administration induced a decrease in the number of dendritic cells in ImiP, and at the time of writing this manuscript, we did not find any reports of this phenomenon. We should also note that there are reports in the literature showing that ImiP has antibiotic effects on *Escherichia coli* and *Yersinia enterocolitica* [66], antimalarial effects [67], and in high doses administered in liposomes, it achieves eradication of *Leishmania donovani* [27]. However, at the time of writing this work, there were only reports indicating that ImiP had no effect on Brucella in vitro [38]. Our results suggest that the effect of this TCA may be due to macrophage activation.

Our results show that the mice in the ImBa groups had equivalent IFN-γ levels to those in the infected group during follow-up. This apparent dissonance could be explained by the elevated levels of extracellular serotonin detected at 14 days. These were associated with ImiP administration and could have triggered the release of IFN-γ [68], whose levels have been associated with increased efficiency of phagocytic cells to control infection [69]. Such a hypothetical scenario may be feasible in the context of our experiments, since IFN-γ released by NK cells [68] could have activated splenic macrophages in addition to the activation induced by ImiP, as previously discussed, thus decreasing the number of *B. abortus 2308* CFU in the spleen. This fact partially explains the difference in the intensity of the phenomenon. There was a reduction in CFU because IFN-γ levels had decreased in both the ImBa and Ba groups by day 28 of follow-up; however, a greater number of splenic macrophages was observed.

Our data showed a significant decrease in IL-6 levels in ImBa mice; a similar fact has been described previously, as ImiP administration reduces the secretion of IL-1β, TNF-α and IL-6 by macrophages [30]. IL-6 has multiple biological effects, not all of which are beneficial [55]. This inflammatory molecule may act as a trigger to initiate a neuropathic pain process through the ubiquitous glycoprotein gp130, which serves as its receptor or is associated with IL-6R and could be localized in nociceptor endings [70]. The increase in IL-6 levels favors the upregulation of vanilloid receptors and ankyrin-1 in neuronal endings and the release of calcitonin gene-related peptide (CGRP) and substance P (SP), among others [54,55]. This peripheral sensitization mediated by IL-6 and gp130 promotes an increase in cytokines and proteases released by microglia and astrocytes in nerve ganglia due to the increase in neurotransmitters, adenosine prostaglandins, and nitric oxide [55]. All these mediators produce a prolonged hyperalgesia, which could partially explain the fact that the mice of the Ba group, which had high levels of IL-6, showed a significant decrease in equilibrium and muscle strength. On the other hand, it has been described that the consumption of ImiP induces a reduction of circulating levels of IL-6 [25,56]. A decrease in IL-6 improved the physical performance of infected animals.

In this work, we have presented data suggesting that the use of ImiP is likely to improve the clinical outcome of mice infected with *B. abortus 2308* (Table 2). It seems fair to say that when used at low doses (≈two times lower than the maximum dose a human can receive; [71]), ImiP increases the psychoimmune resilience of the individual and prevents adverse effects in the liver and kidney [25,26].

### Limitations

Like most experimental studies, the present work has limitations that must be commented on. One such limitation is the use of orogastric tubes for the administration of ImiP, which could have stressed the animals, although it is the only way to standardize the consumption of the administered dose. Further studies should also consider the effect of IL-1 on serotonin levels in the hippocampus, which has been reported in other infectious models and in vivo assays because of lipopolysaccharide or IL-1β administration. It would also be interesting to evaluate the function of macrophages and dendritic cells of the ImBa group in future studies using phagocytosis and reactive oxygen species production assays. A broader battery of behavioral tests should be implemented to assess psychoimmune resistance in depth and that adapted to the biosafety cabinet space (e.g., pain).

## 4. Materials and Methods

### 4.1. Mice

Six- to eight-week-old female BALB/c mice (*n* = 80; 18–21 g body weight) supplied by Ferandhel, Mexico, were used in the study and housed in groups of five per cage. Upon arrival, the mice were allowed to acclimate for 21 days before the start of the experiments. They were kept in temperature (21 °C)- and light (7:00 on/19:00 off, regular cycle)-controlled rooms and housed in cages with microfilters at the animal facility of the Department of Immunology of the Escuela Nacional de Ciencias Biológicas (ENCB), Instituto Politécnico Nacional. The mice had free access to sterilized water and food (Lab Rodent Diet 500, Minneapolis, MN, USA) always. All procedures, such as water, food, and bedding changes, were performed in a biosafety cabinet (Nuaire Class II Type A/B3) to avoid contamination with external agents. The study design included eight groups of 10 mice each, as shown in Figure 1. Day 0 of the study was considered when mice received PBS or *B. abortus 2308* intraperitoneally (IP). In the groups receiving imipramine, imipramine administration started on day 8 after IP administration of *B. abortus 2308* or PBS. In all cases, mice were sampled at day 14 or 28 post-IP administration; four groups were followed for 14 days, and the remaining ones were followed for 28 days. Groups were identified as follows: 14- and 28-day controls (Ctr14 and Ctr28); 14- and 28-day *B. abortus 2308*-infected mice (Ba14 and Ba28); 14- or 28-day *B. abortus* 2308-infected and 6- or 20-ImiP-treated mice (Im6Ba14 and Im20Ba28); and 6- and 20-day ImiP-treated mice (ImiP6 and ImiP20). On day 0, Ctr14, Ctr28, ImiP6, and ImiP20 received a single intraperitoneal administration of either phosphate buffer saline (PBS; 0.1M, pH 7.4; 100 μL) or 1 × 10^6^ colony-forming units (CFU) of *B. abortus 2308* suspended in 100 μL PBS inside a biosafety cabinet (Nuaire Class II Type A/B3). Behavioral tests (see below) were performed, and blood samples were collected inside a biosafety cabinet on days 14 or 28 after IP infection/inoculation. All mice were then sacrificed by cervical dislocation, and brain samples were dissected (see below). Animal handling and experimental protocols were reviewed and approved by the local ethics committee (Registry No. ENCB/CEI/077/2020).

### 4.2. Imipramine Administration

ImiP tablets (25 mg; Psicofarma, Mexico City, Mexico) were ground and dissolved in sterile water to the required concentration of 15 mg/kg/day [72]. ImiP (0.3 mg in 100 µL) was administered via an orogastric tube (Cat: FTP-20-38; Instech, Plymouth Meeting, PA, USA) to ImiP6 and Im6Ba14 mice from days 8 to 14 and to ImiP20 and Im20Ba28 mice from days 8 to 28.

### 4.3. Behavioral Test

On day 14 or 28 post-IP administration, all mice were subjected to a battery of modified tests that we have previously reported [18]. The tests were as follows: (i) forelimb grip strength test (FGST); (ii) motor and balance coordination test (MBCT); (iii) open-field test (OF); (iv) forced swim test (FST); and (v) tail suspension test (TST). Grip strength in the FGST was estimated by pulling the tail while the subject held on to a dynamometer (Labessa, Cat. DI1900) until release; the test was performed 5 times in each mouse. For MBCT, mice were placed on a beam (50 cm long, 2 cm wide) 30 cm above the floor and the time taken to cross the beam (latency) was recorded. OF was performed in a 10 × 30 × 5 cm wooden box with the floor divided into nine quadrants. Mice were placed in the central quadrant, exploratory behavior was filmed for 5 min, and the number of quadrant crossings was recorded. For FST, mice were placed in a 1.7 L circular pool warmed to 35 °C, filmed for 5 min, and immobilization was recorded. Both OF and FST are designed to assess motivation. Finally, for TST, mice were suspended by the tail and immobilization was recorded for 5 min. MBCT and FGST assessed the physical performance of the animals, while OF, FST, and TST assessed anxiety and hopelessness. All tests were conducted in a biosafety cabinet.

### 4.4. Collection of Serum, Spleen, and Brain Samples

On the day of completion of clinical follow-up, blood samples were collected from the facial vein of the mice and centrifuged (2000× *g*) at room temperature for 10 min. Serum was then collected, aliquoted (100 μL), and stored at −80 °C until use. Mice were then subjected to cervical dislocation before decapitation. The brain was removed from the skull, and the hippocampus was dissected as previously described [18]. Briefly, the skull was cut with scissors to expose the brain, and the cerebellum and olfactory bulbs were removed. The brain was placed in a ventral position to remove the thalamus and midbrain and to expose the hippocampus and separate it from the cortex. Tissue samples were weighed on an analytical balance, frozen in liquid nitrogen, and stored at −80 °C until further processing.

### 4.5. Determination of Spleen Brucella Colony-Forming Units (CFU)

On the day of completion of clinical follow-up, the spleen was mechanically removed, minced in a Petri dish filled with 5 mL PBS (1M), and passed through a cell strainer (Cat: 431750; Corning^®^, Corning, NY, USA). The resulting cell suspension was washed once in PBS (3 mL), centrifuged, and resuspended at room temperature. A total of 20 µL of the cell suspension was then taken and sequentially diluted (1:10) in 1M PBS to a final dilution of 10^−4^. Then, 20 µL samples of each dilution were plated in duplicate on tryptic soy agar (TSA) for 48 h at 37 °C. The number of Brucella CFU was estimated by multiplying the number of colonies by the inverse dilution factor.

### 4.6. Dendritic Cells and Macrophages Determination in Flow Cytometry

Ammonium chloride potassium (ACK) buffer was used to eliminate erythrocytes from the spleen cell suspension. The cell suspension was then adjusted to 1 × 10^6^ cells and incubated with the following antibodies for 20 min: PE anti-mouse MHC II(I-A/I-E), negative lineage (PerCP-Cy5.5 anti-mouse CD19, CD3, and B220) (BioLengend, San Diego, CA, USA), PE-CF594 anti-mouse CD11c, and V500 anti-mouse CD11b (BD Bioscience, San Jose, CA, USA). Cells were washed with PBS and analyzed on a FACS-Aria III cytometer (BD Bioscience, San Jose, CA, USA) on the same day of collection for a total of one million events. Autofluorescence and compensation controls were used for each fluorochrome. The analysis strategy was as follows: detritus and double events were eliminated by comparing area to size forward scatter; then, granulocytic cells were selected in a plot of side scatter versus forward scatter. From this region, CD11c+, I-A/I-E+ double-positive cells were selected. Macrophages were CD11b-positive and dendritic cells were Lin-, MHC-II+, and CD 11c+ (see Supplementary Figure S1).

### 4.7. Cytokine Quantification

Serum samples were used to estimate IL-6, IL-12, TNF-α, IFN-γ, MCP-1, and IL-10 concentrations using a mouse inflammation kit (BD™ catalog # 552364), according to the manufacturer’s instructions. Samples were analyzed on a FACSARIA III flow cytometer (BD Bioscience, San Jose, CA, USA). The manufacturer’s limits of detection for cytokines were as follows: 5 pg/mL for IL-6; 17.5 pg/mL for IL-10; 52 pg/mL for MCP-1; 2.5 pg/mL for IFN-γ; 10.7 pg/mL for IL-12; and 7.3 for TNF-α.

### 4.8. Serotonin Quantification by High Performance Liquid Chromatography (HPLC)

#### 4.8.1. Hippocampal Tissue Processing

The hippocampus was homogenized via sonication (≈5 mg tissue) in 200 μL serotonin (5HT) extraction buffer containing 5% citric acid, 200 mM sodium phosphate, 2.5 mM L-cysteine, and 2.5 mM EDTA. Then, 50 μL of 0.4 M perchloric acid was added to precipitate proteins after incubating the homogenate at 20 °C for 20 min. Finally, homogenates were centrifuged at 12,000× *g* at 4 °C for 10 min, and the supernatants were collected, aliquoted (200 μL), and stored at −80 °C until used.

#### 4.8.2. Solid Phase Extraction (SPE) of Serotonin

Serotonin concentration was enriched using a solid phase extraction (SPE) column (Octadecyl C_18_, 1 mL, Cat. 702001; J.T. Baker). Briefly, we used 0.1% trifluoracetic acid (TFA) in water and 0.1% TFA in acetonitrile as mobile phase A (MP-A) and B (MP-B), respectively. The column was then loaded with 250 μL supernatant, followed by a wash with 250 μL MP-A. Finally, 250 μL MP-B was used to elute serotonin.

#### 4.8.3. Chromatographic Runs

Serotonin chromatograms were obtained via reversed-phase HPLC (RP-HPLC) on a JASCO HPLC system (PU-2089 plus pump, AS-2057 plus autosampler, and X-LC™3120FP fluorescence detector; Jasco, Inc. Easton, MD, USA). The system was controlled by ChromNav (Jasco, Inc., Easton, MD, USA). The column (CAPCELL PAK C_18_ column, 300 Å, 5 μ, 4.6 × 250 mm, Shiseido^®^, Cat. 92533) was equilibrated with 100% MP-A; then, 90 μL of each sample was injected in duplicate. All runs were performed with at a column temperature of 30 °C. Chromatographic runs were performed in a linear gradient as follows: 0–3 min 100% MP-A; 3–33 min 0–10% MP-Bl; 33–38 min 10–20% MP-B; and 38–39 min 20–30% MP-B. Then, 30% MP-B was maintained until 43 min to clean the column, and at 43–45 min, the column was switched back to 100% MP-A. Finally, 100% MP-A was maintained until 60 min to equilibrate the column for the next injection. The serotonin peak was detected at min 26 with the following detector parameters: gain, 100; attenuation, 32; response, 20 s; excitation, 280 nm; and emission, 315 nm.

### 4.9. Statical Analysis

All statistical analyses were performed with GraphPad Prism, version 10.0.0, for Windows (GraphPad Software, San Diego, CA, USA). Datasets were subjected to the D’Agostino and Pearson omnibus normality test. In all cases, if the distribution was normal, Welch’s ANOVA test was used, and the value of W was described in the text. When the distribution was non-Gaussian, the Brown–Forsythe ANOVA test was used, and the F* value was described in the text. In both cases, the Dunnett T3 post hoc test was used. In Figure 2, Figure 3, Figure 4 and Figure 5, the following color coding is used: black lines represent comparisons between days 14 and 28 in the same group; red lines indicate when any group is compared to ImBa groups; green lines indicate when Ba groups are compared to Ctr groups; and blue lines are used when ImiP groups are compared to Ctr groups. In all cases, the scatter plot shows the mean ± SD values. Significance was determined when *p* < 0.05.

## 5. Conclusions

Orogastric administration of imipramine reduced serum IL-6 levels and bacterial counts in the spleen, restored serotonin levels in the hippocampus to levels equivalent to controls, increased motivation scores, and improved physical fitness in the subjects. The results suggest that treatment with imipramine for 6 days is more effective. Thus, imipramine may be a pharmacological agent worth considering as an addition to the pharmacological options for the treatment of brucellosis as it enhances the immune response and psychoimmune resilience at a safe dose.

## Figures and Tables

**Table 1:** Cytokine serum levels.

	Groups								Post Hoc Analysis							
Cytokine	Cr14	Ctr28	Ba14	Ba28	Im6Ba 14	Im28Ba 28	ImiP6	ImiP20	Ctr14 vs. Ba14	Ctr28 vs. Ba28	Ba14 vs. Ba28	Ba14 vs. ImBa 20	Ba28 vs. Im20Ba28	Im20Ba28 vs. Im6Ba 14	Im6Ba 14 vs. Ctr14	Im20Ba 28 vs. Ctr28
IL-6	ND	ND	18.6 ± 1.4	12.4 ± 1.5	10.4 ± 2.9	ND	ND	ND	--	--	****	****	--	--	--	--
IL-12	ND	ND	15.1 ± 6.7	10.1 ± 2.7	16.8 ± 4.4	9.8 ± 3.6	ND	ND	--	--	ns	ns	ns	*	--	--
TNF-α	13.9 ± 1.5	13.4 ± 1.5	40.8 ± 6.6	26.9 ± 4.7	29.9 ± 2.7	7.9 ± 11.8	12.5 ± 2.2	14.1 ± 1.9	****	****	**	**	****	****	****	****
IFN-γ	ND	ND	244.8 ± 29.6	64.5 ± 15.1	238.0 ± 29.4	65.9 ± 15.2	ND	ND	--	--	****	ns	ns	****	--	--

Differential statistical tests were used to determine the significance of the values. Brown–Forsythe ANOVA test was used for TNF-α, and Welch’s ANOVA test was used for IL-6, IL-12, and IFN-γ. This table shows cytokine quantification at days 14 and 28 of follow-up in control mice (Ctr14 and Ctr28), ImiP mice (ImiP6 and ImiP20), *B. abortus 2308*-infected mice (Ba14 and Ba28), and ImiP-treated *B. abortus 2308*-infected mice (Im6Ba14 and Im20Ba28). Statistical significance is shown as follows: * = *p* < 0.05; ** = *p* < 0.1; and **** = *p* < 0.0001. ND = not detectable. ns = no significant.

**Table 2:** Effects of imipramine in *B. abortus 2308*-infected mice.

Variable	Effects of Imipramine (6) at Day 14 of Follow Up	Effects of Imipramine (20) at Day 28 of Follow Up
Decrease of hopelessness (FST and TST)	✓	✓
Decrease of anxiety (OF)	✓	±
Increase of serotonin in hippocampus	✓	±
Increase of motor abilities (FGST and MBCT)	✓	✓
Decrease of M and DC in splenic CFU	✓	✓
Decrease of IL-6 serum levels	✓	✓
Decrease of TNF-α serum levels	✓	✓

Effects of imipramine on variables assessed in infected mice. The number in parentheses (6 or 20) indicates the number of days imipramine was administered. ✓ indicates that imipramine restores the values of the variable to levels like those of the control group. ± indicates that imipramine restores the values of the variable, but not like those of the control group. serotonin: serotonin; FST: forced swimming test; TST: tail suspension test; OF: open field; MBCT: motor balance and control test; FGST: forelimb grip strength test; M: macrophages; DC: dendritic cells; CFU: colony-forming unit.

## Data Availability

Data is contained within the article and Supplementary Material.

## Supplementary Materials

The following supporting information can be downloaded at https://www.mdpi.com/article/10.3390/ph16111525/s1: Figure S1. Flow cytometry analysis strategy; Table S1. Mean ± SD of open field, tail suspension test, forced swimming and serotonin; Table S2. Mean ± SD of motor balance and control test and grip strength test; Table S3. Mean ± SD of macrophages and dendritic cells in spleen; Table S4. Mean ± SD of *Brucella abortus 2308* in spleen.
