# Supplementary material for: Imipramine Administration in Brucella abortus 2308-Infected Mice Restores Hippocampal Serotonin Levels, Muscle Strength, and Mood, and Decreases Spleen CFU Count
Source: Pharmaceuticals (Basel). 2023 Oct 27;16(11):1525. doi: 10.3390/ph16111525 (PMC10674296; doi:10.3390/ph16111525)
Supplement: Supplementary file 1 [file pharmaceuticals-16-01525-s001.zip › pharmaceuticals-2597090-supplementary.pdf]

Supplementary Material

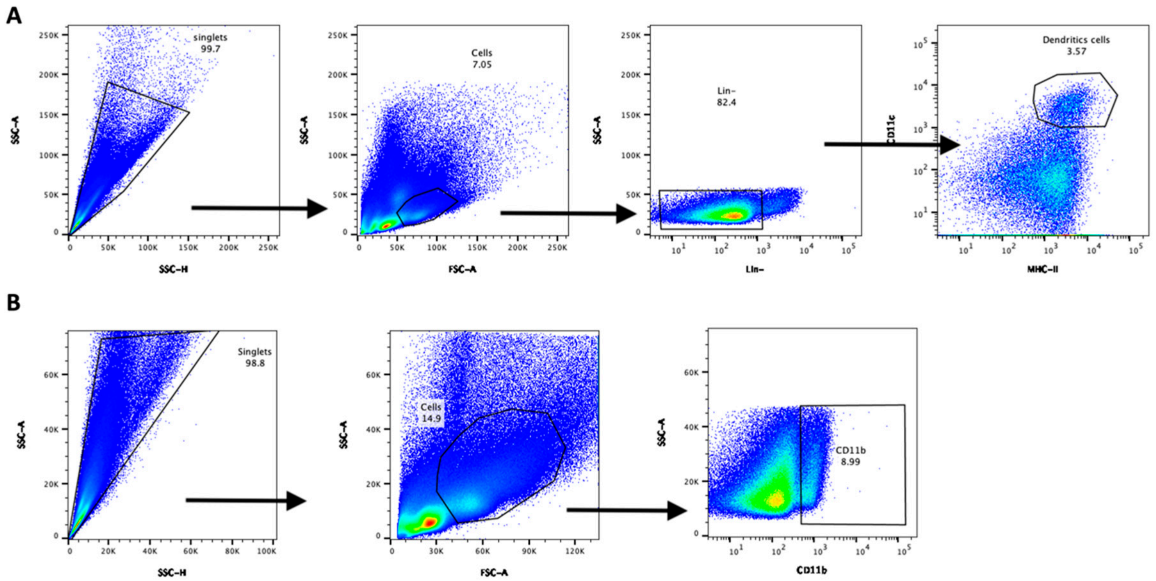

Figure S1. Flow cytometry analysis strategy. Gating used for analysis of macrophages (A) and dendritic cells (B).

Table S1. Mean ± SD of open field, tail suspension test, forced swimming and serotonin

Group	Open field (Number of quadrants crossings)	Tail suspension test Time of immobility (s)	Forced swimming test Time of immobility (s)	Serotonin pg/mg of brain tissue
**Ctr14**	43.70 ± 5.39	66.90 ± 8.81	74.40 ± 3.20	14.18 ± 1.62
**Ctr28**	40.00 ± 4.87	65.60 ± 6.80	65.33 ± 7.08	12.93 ± 1.58
**Ba14**	14.40 ± 3.37	133.70 ± 21.74	130.90 ± 13.23	6.62 ± 0.71
**Ba28**	26.90 ± 3.92	155.20 ± 20.71	155.00 ± 21.56	9.83 ± 0.90
**Im6Ba14**	47.30 ± 4.27	67.60 ± 8.43	99.40 ± 17.09	15.46 ± 3.35
**Im20Ba28**	22.80 ± 7.00	77.60 ± 20.30	75.60 ± 20.60	10.79 ± 1.62
**ImiP6**	60.70 ± 7.30	70.10 ± 10.63	81.00 ± 14.49	15.23 ± 2.46
**ImiP20**	56.20 ± 10.88	71.60 ± 7.07	74.67 ± 5.95	12.23 ± 0.81

The table shows the mean ± SD of the values obtained in the groups analyzed. Open field measure the number of quadrant crossings, a lower number of quadrants crossing can be interpreted as anxiety. Tail suspension test and forced swimming evaluates hopelessness, a higher time of immobility indicates increased hopelessness. Serotonin was quantified in hippocampus.

Table S2. Mean ± SD of motor balance and control test and grip strength test

Group	Motor Balance and Control Test Time (s)	Forelimb Grip Strength Test Newtons (N)
**Ctr14**	17.20 ± 2.09	0.45 ± 0.04
**Ctr28**	17.30 ± 0.94	0.45 ± 0.02
**Ba14**	28.40 ± 3.20	0.30 ± 0.03
**Ba28**	25.90 ± 1.37	0.33 ± 0.04
**Im6Ba14**	18.50 ± 2.79	0.43 ± 0.07
**Im20Ba28**	21.00 ± 1.49	0.49 ± 0.03
**ImiP6**	19.70 ± 2.05	0.45 ± 0.05
**ImiP20**	18.22 ± 1.56	0.48 ± 0.04

The table shows the mean ± SD of the values obtained in the groups analyzed. The motor balance and control test measure the time it takes the mouse to perform the test; a long time can be interpreted as a motor disability. The forelimb grip strength test evaluates the muscular endurance of a mouse in Newtons; a lower value in the test indicates an increase in muscle weakness.

Table S3. Mean ± SD of macrophages and dendritic cells in spleen

Group	Number of macrophages per 1x10^5 splenocytes	Number of dendritic cells per 1x10^5 splenocytes
**Ctr14**	612.80 ± 144.30	120.10 ± 16.35
**Ctr28**	624.20 ± 85.19	134.30 ± 24.36
**Ba14**	1797.00 ± 396.60	277.30 ± 29.94
**Ba28**	4446.00 ± 1279.00	387.00 ± 84.94
**Im6Ba14**	1509.00 ± 325.60	269.20 ± 53.99
**Im20Ba28**	2820.00 ± 1482.00	190.90 ± 35.37
**ImiP6**	643.60 ± 147.50	62.60 ± 18.73
**ImiP20**	783.00 ± 107.70	60.40 ± 20.80

The table shows the mean ± SD of the values obtained in the groups analyzed. Macrophage and dendritic cell counts were performed in the spleen and an adjustment was made to make a ratio of cells per 1x105 splenocytes.

Table S4. Mean ± SD of *Brucella abortus 2308* in splenn

Group	*Brucella abortus 2308* CFU in spleen
**Ba14**	2720460 ± 845314
**Ba28**	26457 ± 13078
**Im6Ba14**	289706 ± 95787
**Im20Ba28**	3377 ± 1272

The table shows the mean ± SD of B. abortus 2308 Colony Forming Units (CFU) derived from individual spleens in B. abortus 2308 infected mice (Ba) and ImiP-treated, B. abortus 2308 infected mice (ImBa)
